# Debridement Arthroplasty for Post-traumatic Stiff Elbow: Intraoperative Factors Affecting the Clinical Results of Surgical Treatment

**DOI:** 10.4055/cios.2009.1.1.27

**Published:** 2009-02-06

**Authors:** Yong Girl Rhee, Nam Su Cho, Chan Teak Lim, Jin Woong Yi

**Affiliations:** Department of Orthopaedic Surgery, School of Medicine, Kyung Hee University, Seoul, Korea.; * Department of Orthopaedic Surgery, Kyung Hee University, East-West Neo Medical Center, Seoul, Korea.; † Department of Orthopaedic Surgery, School of Medicine, Konyang University, Daejeon, Korea.

**Keywords:** Elbow, Stiffness, Debridement arthroplasty

## Abstract

**Background:**

This study evaluated the outcomes of debridement arthroplasty for stiff elbows, as well as the factors affecting clinical outcomes after surgical treatment.

**Methods:**

Eighteen patients with post-traumatic stiff elbows were treated with debridement arthroplasty using a posterior approach. The mean patient age was 33 years (range, 16 to 59 years), and the average follow-up period was 59 months (range, 24 to 141 months). The patient's ability to perform activities of daily living, including combing their hair, feeding themselves, performing hygiene, and putting on shirt and shoes, were evaluated using the Mayo Elbow Performance Score.

**Results:**

At the last follow-up, 16 elbows had painless motion. Two patients continued to complain of mild intermittent pain. The flexion and extension improved to 121° and 10° after surgery, respectively, indicating an average 34° increase in elbow flexion range and an average 25° increase in elbow extension range (*p* < 0.001, *p* < 0.001). The Mayo Elbow Performance Score at the last follow-up was excellent in nine elbows (50%) and good in nine elbows (50%).

**Conclusions:**

Debridement arthroplasty is a predictable procedure for the treatment of intractable stiff elbow, provided that the elbow is stable and congruous.

Post-traumatic stiff elbow is a rather common disorder that develops after dislocation of the elbow with or without fracture, or after a fracture of the distal humerus, particularly with intra-articular involvement. Fractures of the olecranon and radial head can also cause stiff elbows. Limited motion of the elbow often occurs after severe damage to the peri-articular soft tissues.1) Unfortunately, no large joint other than the elbow is as prone to post-traumatic stiffness. Once post-traumatic stiffness develops around the elbow joint, it is difficult to restore mobility, which is the key to the success of all procedures. Failure to gain full mobility of the elbow often leads to functional disability, resulting in the need for surgical intervention.

Several surgical options have been introduced for the treatment of stiff elbows after conservative treatment has failed. One option is arthroscopic release.^2^,3) However, this technique has limited applicability for the treatment of severely stiff elbows. When the elbow joint maintains its congruity, with both anterior and posterior components involved, predictable results can be achieved with a columnar procedure, ulnohumeral arthroplasty, or debridement arthroplasty, the latter of which is an extensive procedure.^4^-9)

This paper reports the clinical outcomes of open debridement arthroplasty in post-traumatic stiff elbows and describes the factors affecting these outcomes.

## METHODS

Between June 1991 and June 1999, the senior author treated 18 patients with post-traumatic stiff elbows using posterior approach debridement arthroplasty. The mean patient age at the time of surgery was 33 years (range, 16 to 59 years). There were 11 men and 7 women. The mean follow-up period was 59 months (range, 24 to 141 months), and the mean duration of symptoms before the procedure was 13 months (range, 5 to 36 months). Prior injuries included intra-articular fracture of the distal humerus in six patients, olecranon fracture in six, elbow dislocation in three, severe injury of the soft tissues in two, and a radial head fracture in one. Patients with primary osteoarthrosis, heterotopic ossification around the elbow, stiffness with nonunion or malunion of the elbow joint, or articular incongruity were excluded. In addition, patients who had undergone previous surgical procedures were also excluded.

### Preoperative Evaluation

The level of preoperative pain was mild in four patients, moderate in nine, and severe in five, according to the Mayo Elbow Performance Score.10) The mean points of maximum flexion and extension and the total flexion-extension arc were 86.8°(range, 40° to 130°), 35°(range, 10° to 60°), and 51.9° (range, 0° to 100°), respectively. The mean points of maximum pronation and supination and the total rotation arc were 58.1° (range, 0° to 75°), 61.9° (range, 0° to 85°), and 120° (range, 0° to 150°), respectively (Table 1). The total flexion-extension arc was < 30° in four patients, 31° to 60° in eight, 61° to 90° in five, and > 90° in one. All had a combined contracture. The preoperative Mayo Elbow Performance Score10) for motion was 10.6 (Table 2). Ten elbows (56%) showed symptoms associated with ulnar nerve entrapment. All elbows were stable. The patients' ability to perform activities of daily living, such as combing their hair, feeding themselves, performing hygiene, and putting on shirt and shoes, was evaluated using the Mayo Elbow Performance Score.10) Preoperatively, the patients were able to perform a mean of 3.1 of the 5 daily activities. Among these, hygiene was the least difficult activity (3.9 ± 2.1), while combing hair and putting on shoes were the most difficult (2.2 ± 2.6) (Table 3). The function score was 15.6, and the overall score was 50.2 (Table 2). The preoperative function was 14 in the elbows with ulnar nerve symptoms and 17.5 in the elbows without preoperative entrapment symptoms of the ulnar nerve.

### Operative Technique

**Fig. 1:**
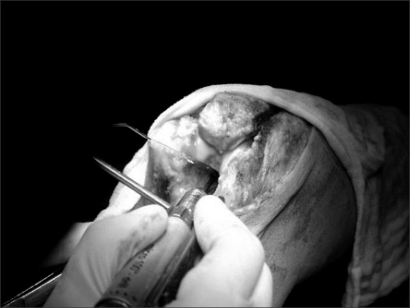
The olecranon fossa was fenestrated using an electric burr because the osteotomized tip of the olecranon had encroached on the osteophytes of the olecranon fossa.

**Fig. 2:**
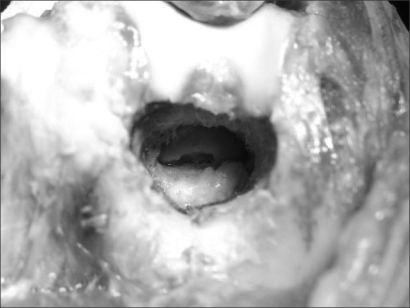
Anterior capsular release and coronoid excision were carried out through the hole.

**Fig. 3:**
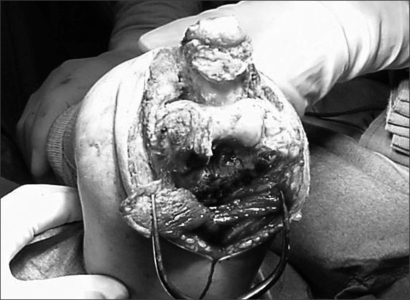
The collateral ligament was released in a patient with elbow flexion limitation.

Each patient was placed in the supine position under general anesthesia. The arm was draped free and brought across the chest. After a posterior approach was achieved through a curvilinear incision, the triceps in continuity with the periosteal sleeve over the olecranon was reflected from the medial side. The ulnar nerve was identified and prepared for transposition. Subcutaneous ulnar nerve transposition was performed in 15 elbows. Ten patients had shown ulnar nerve symptoms, and five had exhibited a < 90° range of motion (ROM) limitation in the elbow. After wide joint exposure was achieved, the tip of the olecranon was osteotomized, and all loose bodies from the olecranon fossa were removed. Osteophytes around the olecranon were excised. The olecranon fossa was then fenestrated using an electric burr in ten elbows, because the osteotomized tip of the olecranon had impinged the osteophytes of the olecranon fossa (Fig. 1). The fenestrated hole was oriented slightly distal to the olecranon fossa to accommodate the distal tip of the olecranon, and the edge of the hole was made slightly larger than the concavity of the original fossa. The elbow was then flexed, which brought the coronoid process into view through the fenestration hole. Anterior capsular release and coronoid excision were performed through the hole (Fig. 2). The collateral ligament was released in patients with limited elbow flexion because the posterior band of the medial collateral ligament was not isometric (Fig. 3). The collateral ligament was released in eight elbows. Release of the collateral ligament is recommended because intraoperative ligamentous disruption or manipulation-induced bony avulsion may develop. Fenestration was not performed if the osteophytes from both the olecranon and the coronoid process were less severe. Instead, only the olecranon tip was trimmed from the posterior aspect, and the elbow was dislocated to sufficiently release the anterior capsule. An additional excision of the olecranon tip or more extensive release of the anterior capsule was required if the extension was inadequate.

### Rehabilitation

**Fig. 4:**
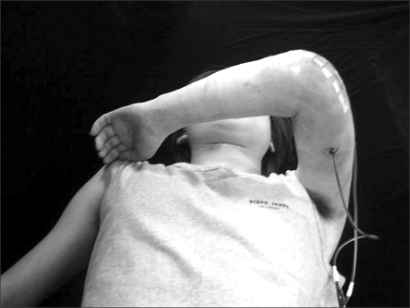
Passive extension motion and early overhead down exercise for flexion began one day after surgery

A hyperextension splint was applied on the fourth day after surgery and replaced by an alternative splint after the swelling had subsided. A flexion splint was applied during the day after passive motion, and a hyperextension splint was worn at night. Passive extension motion and early overhead down exercise for flexion began one day after surgery (Fig. 4). Gradually progressive passive flexion up to 90° was easily performed with the patients' cooperation. The so-called "gravity-weight down exercise" was carried out for patients having difficulty flexing their arms more than 90°. With the shoulder flexed 90° and internally rotated in the sagittal plane and the elbow flexed 90°, gravity-weight down exercise allows gravity and the weight of the forearm to gradually bend the elbow. All the patients in this study received oral nonsteroidal anti-inflammatory drugs (NSAIDs) for postoperative pain. Patients were given intramuscular injections of NSAIDs when needed. No drug that prevents ectopic bone formation was administered.

### Statistical Analysis

The Wilcoxon Signed Rank Test and the Mann-Whitney U Test were used for statistical analyses. The SPSS ver. 11.0 was used for all statistical analyses, with the a level set at 0.05.

## RESULTS

Preoperatively, 14 elbows (78%) showed moderate to severe pain. At the last follow-up evaluation, ten patients (56%) reported no pain, and eight patients (44%) reported mild pain. The mean gains in flexion and extension were 34° and 25°, respectively. The mean overall gain in the flexion-extension arc was 59° (Table 1), and the delayed loss of motion at the time of the latest follow-up was 3.6° (range, 0° to 25°). The ROM in rotation remained largely unchanged from the preoperative level. The flexion-extension arc of the eight elbows that had undergone fenestration of the olecranon fossa was 114.8°, compared with 105.6° in the ten elbows that had not. This is because the olecranon tip had not impinged against the olecranon fossa. In this study, the fenestration group showed better flexion-extension arcs, rotation arcs, and function compared to the non-fenestration group (Table 4). The final flexion-extension arc was 108.4° in the eight patients who required release of tightened collateral ligaments, compared with 112.6° in the ten patients who did not. Collateral ligament release did not significantly influence the statistical results of the motion and functional scores. In particular, the controlled partial or complete release of the collateral ligaments had no adverse effect on stability (Table 5). In addition, the patients with elbows that had undergone anterior transposition reported similar final results in terms of pain, motion, stability, and function, compared to the patients without transposition (Table 6). Postoperative function almost returned to normal. Patients were able to perform a mean of 4.5 of the 5 daily activities, up from the preoperative values of 3.1 activities. Among these, the mean value for "feeding oneself", "hygiene", and "putting on a shirt" was 4.7, and the mean value for "combing hair" and "putting on shoes" was 4.2 (Table 3). After surgery, the overall postoperative score increased to 89.4 points, up from 50.2 points. The pain score improved from 14.2 to 38.3 points, while ROM increased from 10.6 to 18.9, and function increased from 15.6 to 22.5. However, there was no significant change in stability. The Mayo Elbow Performance Score10) at the last follow-up was excellent in nine elbows (50%) and good in nine (50%).

## DISCUSSION

Stiffness of the elbow joint is defined as limitation of motion at the end points of the normal arc of flexion and extension. When conservative treatment fails, several options are available on the condition that the articular surface is intact, including arthroscopic or open capsular release, hinge distraction, and debridement arthroplasty.^1^,^3^-^6^,^11^,12) Advanced arthroscopic techniques can be applied to the treatment of stiff elbows. Although the arthroscopic approach is an attractive option with lower morbidity, it has limited indications. The final results are less predictable in elbows with extra-articular stiffness or with a history of arthroscopic capsular release secondary to severe fibrous ankylosis.5) Arthroscopic debridement may be indicated in mild stiffness if there are osteophytes present on the upper margin around the coronoid fossa or on the olecranon tip.^2^,3)

However, in severely stiff elbows, open debridement is more desirable to achieve greater motion than is arthroscopic debridement.5) In the treatment of osteoarthritis of the elbow, Cohen et al.5) reported that open debridement is superior to arthroscopic fenestration of the olecranon fossa for improving range of motion. In their report, only 8 of 26 arthroscopic debridement patients showed a mean gain of 4° in elbow flexion, whereas 12 of 16 patients who underwent the open Outerbridge-Kashiwagi procedure showed a mean gain of 15° in elbow flexion. The columnar procedure,8) which is a limited lateral approach, is safe, easy to perform, reliable, and quite attractive for an extrinsic stiff elbow. However, it is difficult to release a medial component using this procedure, and another posterior approach is required to expose the ulnar nerve. Occasionally, this procedure results in a delayed loss of motion after a period of postoperative improvement. Cohen and Hastings6) modified the lateral approach, which allows for the release of post-traumatic contractures without disrupting the lateral collateral ligament or the origins of the extensor tendon at the lateral epicondyle of the humerus. As a result, total elbow movement improved by a mean of 55° in all 22 patients in their series. Fenestration of the olecranon fossa through a posterior approach, which was originally described by Kashiwagi, has recently been modified by other authors. Morrey1) advocated ulnohumeral arthroplasty (UHA), in which the triceps is reflected rather than split to expose the olecranon fossa, and a trephine is used to remove the osteophytes encroaching on the olecranon and coronoid fossa. Antuna et al.4) reported that the mean arc of flexion-extension improved from 79° preoperatively to 101° at 18 months postoperatively with UHA for primary degenerative arthritis of the elbow. Tsuge and Mizuseki13) introduced a more extensive debridement, in which the ligaments were released and the elbow was subluxated in order to assess the articular surface, release the anterior capsule, and remove the osteophytes. Minami et al.9) reported that 30 of 44 elbows showed minimal or no pain, while 11 had residual symptoms, 8 to 16 years after the Outerbridge-Kashiwagi procedure. The final arc of motion was 90°, which represented a 17° loss over time. Antuna et al.4) reported that 35 (76%) of 46 elbows exhibited little or no pain and 11 exhibited moderate or severe pain after ulnohumeral arthroplasty, but the mean arc of flexion-extension was 101°. In our study, the final arc of motion averaged 110.7°. It is believed that fenestration and debridement arthroplasty, which release the entire capsule, contributed to this result. Stans et al.12) reported that much of the improvement over intraoperative motion had been gradually lost by the time of the final follow-up. In their series, the total arc of motion in 28 elbows with post-traumatic contracture improved from 56° preoperatively to 125° intraoperatively. However, the final total arc of motion an average of 15 months after the surgery was 81°, and the delayed loss of motion was 44°. Their surgical procedure consisted mainly of an anterior capsulectomy, with an additional posterior capsulotomy when a tight posterior capsule was noted to restrict elbow flexion. Therefore, for an elbow with a severe contracture, a posterior approach-rather than an anterior, medial, or lateral approach-is recommended to release the soft tissues of the anterior, posterior, medial, and lateral components. When osseous impingement is present, the offending bony structure needs to be removed, often by excising the tip of the olecranon or by contouring the distal part of the humerus to recreate the coronoid and the olecranon fossa. Whether the Outerbridge-Kashiwagi method or UHA advocated by Morrey14) is employed, the effects of fenestration on the olecranon and the coronoid fossa are well recognized. Although both methods can release the anterior capsule, remove loose bodies, and excise the coronoid process through fenestration, it is difficult to release an entire lateral or medial musculoligamentous structure that is severely contracted. The medial, lateral, and combined approaches described by Oka et al.11) are good methods for releasing the anterior and posterior capsular structures of a stiff elbow, as is the columnar approach. However, capsular release alone cannot resolve the limitation of motion caused by bone block, because osteophytes from the olecranon tip and the coronoid process are likely to collide with the olecranon fossa or the coronoid fossa, even after removal of the osteophytes. Fenestration larger than the size of the original fossa was performed in cases where severe osteophytes from the olecranon tip seriously limited the extension of the elbow or where the osteophytes from the coronoid process blocked flexion. Our intent in doing so was to avoid collision between the osteophytes and the fossa and to prevent further impingement through the redevelopment of body spurs. This study showed that the fenestration group had less delayed loss of motion. This suggests that fenestration of the fossa is quite effective in preventing the reencroachment caused by reforming spurs. Recently, fenestration has been performed routinely with the release of collateral ligaments to prevent further impingement, regardless of the presence of a bony block. The medial collateral ligament is a primary stabilizer of the elbow joint that needs to be preserved unless it is contracted during surgery for the release of a stiff elbow. Sometimes, release is inevitable for the treatment of a severely contracted elbow. When the ligaments are severely contracted, one may experience body avulsion or a ligamentous tear during final manipulation to gain maximum flexion. It is better to release the ligaments, particularly the posterior bundle, from the humeral epicondyle rather than to face such circumstances. Meticulous reconstruction of released ligaments can achieve stability if they are carefully reattached. Morrey1) reported that instability was not a problem in 26 elbows with post-traumatic contractures, with the exception of one case of infection and three cases of spontaneous joint resorption, even though the joint surfaces had been distracted by 5 mm for three or four weeks with a distraction external device. In the current series, all the ligaments in the eight cases were cut from the humeral side and replaced. Only one patient (who had the radial head excised) complained of instability. Rehabilitation after surgery has particular importance for preventing stiffness, especially in the elbow joint. Sustained joint exercises immediately after the procedure are essential for maintaining the intraoperative range of motion. In order to achieve further flexion with ease, gravity-weight down exercises are recommended, rather than simple passive flexion exercises. Gravity-weight down exercise avoids unnecessary muscle guarding during exercise and allows gravity and the weight of the forearm to provide automatic flexion.

This study was effective in the sense that it was a comparative analysis of additional procedures in the setting of a single disease entity, and that the procedures were performed by a single surgeon at a single institution. However, this study examined a limited variety of procedures and a small number of cases.

In summary, debridement arthroplasty is a predictable procedure for treating an intractable stiff elbow, provided the elbow is stable and congruous. However, meticulous surgical technique and well-programmed rehabilitation are required. This study suggests that a fenestration procedure can avoid further impingement of the anterior and posterior components and prevent a delayed loss of motion. In addition, the collateral ligaments may need to be released partially or completely in order to achieve adequate elbow motion in patients with more severe contractures. Ulnar nerve transposition is also recommended before debridement arthroplasty is undertaken in stiff elbows.

## Figures and Tables

**Table 1:**
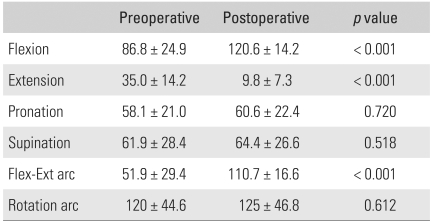
Preoperative and Postoperative ROM (Degrees)

Flex-Ext: Flexion-extension

**Table 2:**
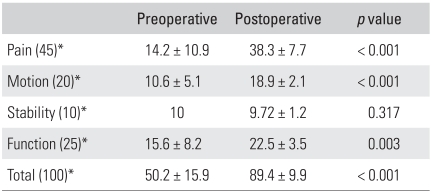
Clinical Outcomes after Debridement Arthroplasty

^*^The Mayo Elbow Performance Index10)

**Table 3:**
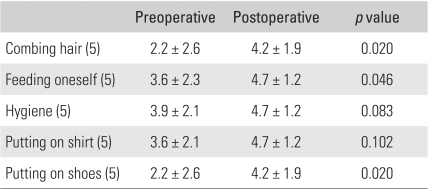
Preoperative and Postoperative Scores for Activities of Daily Living (ADL)^*^

^*^The Mayo Elbow Performance Index10)

**Table 4:**
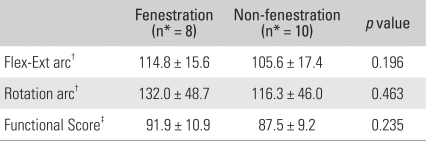
Postoperative Results in the Fenestration and Non-fenestration Groups

Flex-Ext: Flexion-extension, ^*^number of patients, ^†^The values are given as degrees, ^‡^The Mayo Elbow Performance Index10)

**Table 5:**
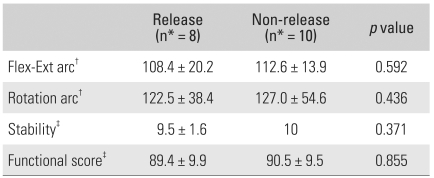
Postoperative Results for Collateral Ligament Release and Non-release Groups

Flex-Ext: Flexion-extension, ^*^number of patients, ^†^The values are given as degrees, ^‡^The Ma-yo Elbow Performance Index10)

**Table 6:**
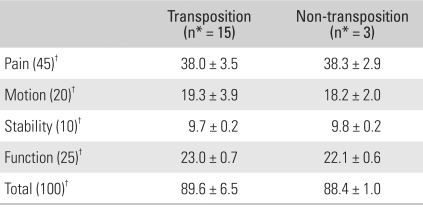
Postoperative Results for the Ulnar Nerve Transposition and Non-transposition Groups

^*^nuber of patients, ^†^The Mayo Elbow Performance Index10)

## References

[1] Morrey BF (1990). Post-traumatic contracture of the elbow. Ope-rative treatment, including distraction arthroplasty. J Bone Joint Surg Am.

[2] O'Driscoll SW (1995). Arthroscopic treatment for osteoarthritis of the elbow. Orthop Clin North Am.

[3] Savoie FH, Nunley PD, Field LD (1999). Arthroscopic mana-gement of the arthritic elbow: indications, technique, and results. J Shoulder Elbow Surg.

[4] Antuna SA, Morrey BF, Adams RA, O'Driscoll SW (2002). Ulnohu-meral arthroplasty for primary degenerative arthritis of the elbow: long-term outcome and complications. J Bone Joint Surg Am.

[5] Cohen AP, Redden JF, Stanley D (2000). Treatment of osteoarthritis of the elbow: a comparison of open and arthroscopic debridement. Arthroscopy.

[6] Cohen MS, Hastings H (1998). Post-traumatic contracture of the elbow. Operative release using a lateral collateral ligament sparing approach. J Bone Joint Surg Br.

[7] Kashiwagi D (1978). Intra-articular changes of the osteoarthritic elbow, especially about the fossa olecrani. J Jpn Orthop Assoc.

[8] Mansat P, Morrey BF (1998). The column procedure: a limited lateral approach for extrinsic contracture of the elbow. J Bone Joint Surg Am.

[9] Minami M, Kato S, Kashiwagi D (1996). Outerbridge-Kashiwagi's method for arthroplasty of osteoarthritis of the elbow: 44 elbows followed for 8-16 years. J Orthop Sci.

[10] Morrey BF, An KN, Morrey BF (2000). Functional evaluation of the elbow. The elbow and its disorders.

[11] Oka Y, Ohta K, Saitoh I (1998). Debridement arthroplasty for osteoarthritis of the elbow. Clin Orthop Relat Res.

[12] Stans AA, Maritz NG, O'Driscoll SW, Morrey BF (2002). Operative treatment of elbow contracture in patients twenty-one years of age or younger. J Bone Joint Surg Am.

[13] Tsuge K, Mizuseki T (1994). Debridement arthroplasty for advanced primary osteoarthritis of the elbow. Results of a new technique used for 29 elbows. J Bone Joint Surg Br.

[14] Morrey BF (1992). Primary degenerative arthritis of the elbow Treatment by ulnohumeral arthroplasty. J Bone Joint Surg Br.

